# The Risk of Drug Interactions in Older Primary Care Patients after Hospital Discharge: The Role of Drug Reconciliation

**DOI:** 10.3390/geriatrics8060122

**Published:** 2023-12-16

**Authors:** Cristina Vocca, Antonio Siniscalchi, Vincenzo Rania, Cecilia Galati, Gianmarco Marcianò, Caterina Palleria, Luca Catarisano, Ilaria Gareri, Marco Leuzzi, Lucia Muraca, Rita Citraro, Giacinto Nanci, Antonio Scuteri, Rosa Candida Bianco, Iolanda Fera, Antonietta Greco, Giacomo Leuzzi, Giovambattista De Sarro, Bruno D’Agostino, Luca Gallelli

**Affiliations:** 1 Operative Unit of Clinical Pharmacology and Pharmacovigilance, Renato Dulbecco University Hospital, 88100 Catanzaro, Italy; cristina_vocca@live.it (C.V.); raniavincenzo1@gmail.com (V.R.); gianmarco.marciano3@gmail.com (G.M.); palleria@unicz.it (C.P.); lucacatarisano@gmail.com (L.C.); ilariagareri1@gmail.com (I.G.); desarro@unicz.it (G.D.S.); 2 Department of Neurology and Stroke Unit, Annunziata Hospital of Cosenza, 87100 Cosenza, Italy; anto.siniscalchi@libero.it; 3 Research Center FAS@UMG, Department of Health Science, Magna Graecia University, 88100 Catanzaro, Italy; galaticecilia86@gmail.com; 4 Department of Primary Care, ASP Catanzaro, 88100 Catanzaro, Italy; marco.leuzzi2@studenti.unicz.it (M.L.); lalumuraca@gmail.com (L.M.); ginanci@libero.it (G.N.); scuteranto@gmail.com (A.S.); rosacandidabianco61@gmail.com (R.C.B.); fera.iolanda@alice.it (I.F.); antoniettagreco@virgilio.it (A.G.); leuzzi.giacomo@tin.it (G.L.); 5 Department of Health Science, Magna Graecia University, 88100 Catanzaro, Italy; citraro@unicz.it; 6 Department of Environmental Biological and Pharmaceutical Sciences and Technologies, University of Campania “Luigi Vanvitelli”, 81100 Caserta, Italy; bruno.dagostino@vanvitelli.it; 7 Medifarmagen SRL, Renato Dulbecco University Hospital, 88100 Catanzaro, Italy

**Keywords:** reconciliation, deprescription, older adult, polytherapy

## Abstract

Introduction: Drug–drug interactions (DDIs) represent an important clinical problem, particularly in older patients, due to polytherapy, comorbidity, and physiological changes in pharmacodynamic and pharmacokinetic pathways. In this study, we investigated the association between drugs prescribed after discharge from the hospital or clinic and the risk of DDIs with drugs used daily by each patient. Methods: We performed an observational, retrospective, multicenter study on the medical records of outpatients referred to general practitioners. DDIs were measured using the drug interaction probability scale. Potential drug interactions were evaluated by clinical pharmacologists (physicians) and neurologists. Collected data were analyzed using the Statistical Package for the Social Sciences. Results: During the study, we evaluated 1772 medical records. We recorded the development of DDIs in 10.3% of patients; 11.6% of these patients required hospitalization. Logistic regression showed an association among DDIs, sex, and the number of drugs used (*p* = 0.023). Conclusions: This observational real-life study shows that the risk of DDIs is common in older patients. Physicians must pay more attention after hospital discharge, evaluating the treatment to reduce the risk of DDIs.

## 1. Introduction

Drug interactions (DDIs) represent an important clinical problem, particularly in patients, due to polytherapy, multimorbidity, and pharmacokinetic changes. A combination of drugs may be useful to manage multimorbidity, but there is a risk of being prescribed potentially inappropriate medications, resulting in adverse drug reactions (ADRs), increased hospitalizations, and higher mortality [1].

A study by Cooper documented that 67.4% of 332 nursing home residents experienced at least one ADR over a 4-year period, related to antipsychotic drugs, nonsteroidal anti-inflammatory drugs (NSAIDs), and insulin [2].

In older people, the diagnosis of ADRs can be difficult because they have nonspecific symptoms, e.g., falls, fatigue, cognitive decline, or constipation, which could be related to several diseases [3].

Failure to recognize an ADR may result in a prescribing cascade whereby a new drug is prescribed to treat the adverse effect of the culprit drug, thus exposing the patient to a continuing risk of ADR from the culprit drug and additional risk from the newly prescribed drug [4,5].

In older patients, comorbidity increases drug consumption and/or prescription, with an increase in the risk of DDIs [6,7].

Poznań and Głogów reported incorrect drug combinations in 28.6% of older patients [8], probably related to the high consumption of OTC drugs (5.5% in incorrect connections). Moreover, in European studies incorrect drug combinations were described in a range between 9.8 and 38.5%, higher than in the United States (21.3–28.8%) [9,10,11], probably due to both differences in the availability of drugs in individual countries and to different practices of prescribing drugs by pharmacists.

Moreover, Roughead et al. [12] reported that about 20% of drugs used by older patients should not be recommended, while this percentage increases in people in nursing homes (30–50% of patients). To reduce the risk of DDIs, drug prescriptions must be evaluated well, taking into consideration other drugs or nutrients that are already being used. Several authors reported that medication errors (e.g., risk of duplication and DDIs) are common upon hospital discharge due to the ignorance of a patient’s current treatment, whereby essential information regarding an outpatient’s medications is omitted [13,14,15].

This study aimed to investigate the association between drugs prescribed during discharge from the hospital or clinic and the risk of DDIs with drugs used daily by each patient.

## 2. Materials and Methods

### 2.1. Study Design

We conducted an observational retrospective study using data recorded on the medical records of outpatients referred to general practitioners of the ASP of Catanzaro (Italy) from 11 August 2017 up to 10 August 2023.

### 2.2. Protocol

Data recorded in clinical records, including age, sex, ADRs (in agreement with the Naranjo probability score), morbidities, polytherapy, and laboratory findings, were analyzed in agreement with previous reports [16,17,18,19].

Information on several medical conditions and other clinical diagnostic events, e.g., cardiovascular and respiratory diseases, diabetes, osteoarthritis, metabolic and rheumatoid disorders, and hospitalizations, were also collected.

Data recorded were evaluated by clinical pharmacologists and neurologists to establish the development of ADRs and DDIs, using both the DIPS (drug interaction probability scale) score and websites: https://intercheckweb.marionegri.it/ (1 September 2023); https://www.drug-pin.com/ (1 September 2023); www.druginteractions.org (1 September 2023); https://janusinfo.se/ (1 September 2023); www.drugs.com (1 September 2023).

The DIPS was used to evaluate the probability of a causal relationship between a potential drug interaction and an ADR. The DIPS estimates the probability that an ADR was caused by an interaction; according to the total score of 10 questions, the relationship is doubtful (<2), possible (2–4), probable (5–8), or highly probable (>8) [20]. The risk of DDIs was defined as follows: A (minor), not clinically relevant; B (moderate), associated with an uncertain or variable event; C (major), associated with a serious event but which can be managed (e.g., by adjusting the dose); D (contraindicated or very serious), associated with a serious event for which co-administration should be avoided or carefully monitored.

### 2.3. Inclusion and Exclusion Criteria

Patients who were 65 years of age or older at the time of their last office visit, had 2 documented visits in the outpatient primary care setting in the previous 2 years, and had been discharged from the hospital in the last year were enrolled. Exclusion criteria included current residence in a skilled nursing facility, Alzheimer’s disease, and active cancer (if cancer was the cause of hospitalization).

### 2.4. Endpoints

The primary endpoint was the number of patients with a drug at risk of drug interactions after hospital discharge. The secondary endpoint was the correlation between ADRs and drug interactions. Another secondary endpoint was the correlation between drug interactions and age, comorbidity, sex, and polytherapy.

### 2.5. Ethical Considerations

This retrospective study was performed on clinical recorders of general practitioners; therefore, written informed consent was obtained from each general practitioner at the time of the first admission to the clinical room. All the procedures were performed according to the Declaration of Helsinki and in accordance with the Good Clinical Practice guidelines. The study protocol was approved by the local Ethics Committee, protocol number 2017/238.

### 2.6. Statistical Analysis

Descriptive statistical analyses were performed to evaluate clinical and demographic characteristics, with continuous data presented as the mean ± standard deviation (SD), while ordinal data were expressed as the percentage. The skewness of continuous variables was assessed using the Kolmogorov–Smirnov test, highlighting non-normally distributed variables. Thus, a non-parametric approach was applied using the Mann–Whitney U test or the Independent-Samples Kruskal–Wallis Test for continuous variables and the two-tailed Pearson chi-squared test or the Fisher’s test for categorical variables as appropriate. A binary regression was used to perform the multivariate analysis. A *p*-value < 0.05 was considered statistically significant. All tests were two-tailed. Statistical analysis was conducted with the Statistics Package for Social Sciences (SPSS) version 26.0 (IBM Corp. SPSS Statistics, Armonk, NY, USA).

## 3. Results

### 3.1. Demographic and Clinical Characteristics

During this study, we retrospectively analyzed 10,596 clinical records. Using the paired sample test, we evaluated a significant difference (*p* < 0.01) between men and women enrolled (men 4647, 43.9%; women 5949, 56.1%), without regard to age (mean age total: 64.4 ± 16.6 years; range 19–100 years; men 64.4 ± 16.6 years; women 63.9 ± 17.8 years).

**Figure 1:**
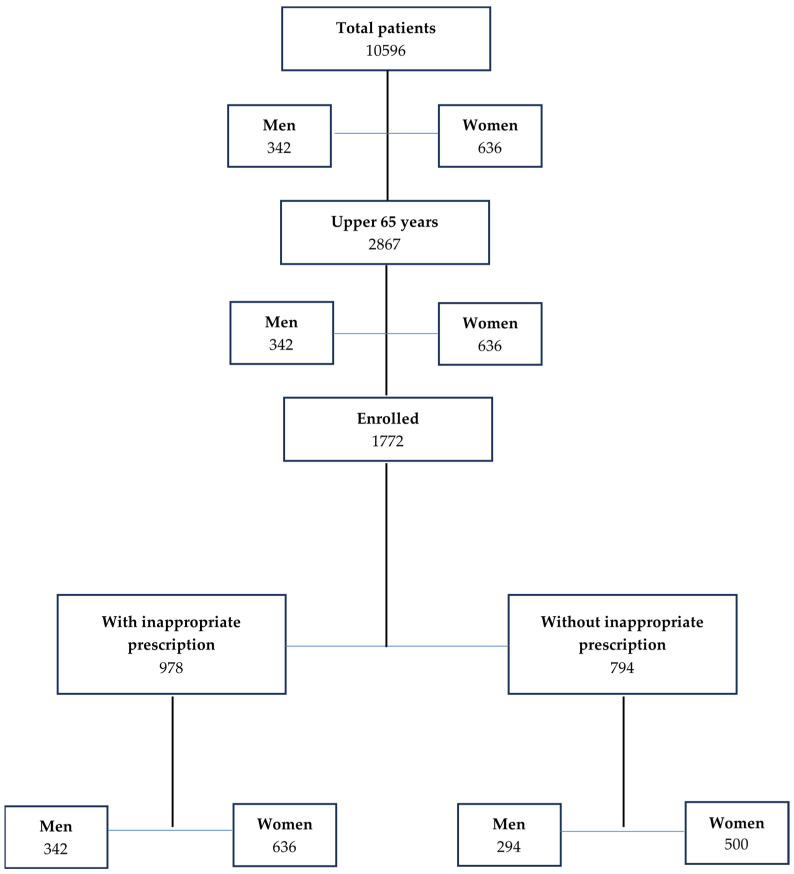
Flow-chart showing the enclosed patients used in this analysis.

After inclusion criteria evaluation, 1772 patients (men 586, 33.1%, women 1186, 66.9%, *p* < 0.01) aged 65 or older were included in this analysis (16.7%) (mean men 77.5 ± 7.7 years, women 77.2 ± 8.2 years, *p* > 0.05) (Figure 1). Of the 1772 enrolled patients, all had at least one morbidity; the most common were cardiovascular diseases (82.3%; including blood hypertension and atrial fibrillation) and osteoarthritis (79.5%) (Table 1).

The mean number of drugs used daily was 6 (range 3–12), with a mean of 5 tablets every day. The demographic characteristics of the enrolled patients are described in Table 2. All included patients showed similar gender characteristics: race (white), geographic region (Calabria, Italy), setting (urban), job (retirees), religion (Catholic).

### 3.2. Inappropriate Drug Prescription

Clinical research performed through both PC software and clinical pharmacologist and neurologist consultants documented that, after hospital discharge (*n* = 1772), 44.8% of the enrolled patients (*n* = 794; *p* < 0.01) received a prescription able to induce a potential DDI (pDDI).

The drugs prescribed after discharge and commonly related to DDIs were omeprazole or esomeprazole (25%) and statins (20%) (Table 3 and Table 4). We also documented that 24 patients (1.8%) developed pDDIs (D Class and C Class) requiring hospitalization due to cardiovascular symptoms (tachycardia, and chest pain), methotrexate toxicity, and muscular pain (Table 5).

In particular, during Lenvatinib treatment, electrocardiographic monitoring, performed in emergency room, revealed a QT > 500 ms, suggesting changing the last drug prescribed during the hospital discharge (Table 5). Moreover, during clopidogrel treatment, the administration of omeprazole or esomeprazole induced the development of confusion, chest pain, and fatigue, probably related cardiovascular impairment, even if no tests were performed to show an association between the DDI and symptoms. Clinical symptoms improved switching the patients from omeprazole or esomeprazole to pantoprazole.

### 3.3. DIPS Score

Using the DIPS, we documented that ADRs were likely induced by DDIs (DIPS score 6–7). It is important to understand that we did not consider the rechallenge and we considered the older adult as a factor able to explain the ADR.

### 3.4. Risk Evaluation

Both univariate and multivariate analysis (binary logistic regression) show that both age and the number of chronic drugs are associated with an increasing risk of DDIs. The adjusted OR increased from 0.9 (95% CI 0.7–1.03) in patients aged 65–75 years to 1.09 (95% CI 1.05–1.22) in patients aged 76–85 years to 1.79 (95% CI 1.56–1.95) in those aged 85 or older. Patients treated with 3–5 chronic drugs have a lower risk (OR = 2.93; 95% CI 2.83–3.08) of pDDIs (OR = 5.69; 95% CI 5.58–5.91) than those receiving more than five chronic drugs. Finally, we documented that women have a statistically significant higher risk of developing pDDIs (OR = 2.85; 95% CI 2.37–3.46) than men (OR = 1.25; 95% CI 1.08–1.42).

## 4. Discussion

In this study we evaluated the risk of drug interactions after hospital discharge in older patients.

The careful monitoring of polytherapy is necessary among older patients, in order to reduce the risk of drug prescriptions that could induce pDDIs. Polytherapy represents a criterion of frailty in older adults and a risk factor for mortality and morbidity due to the increased risk of DDIs, ADRs (risk of falls and cognitive impairment), and greater use of health resources [21].

In this study, we demonstrated that adults aged 65 years and older take an average of five tablets every day with an increased risk of pDDIs. Considering the comorbidities, we documented that all patients have more than one comorbidity, particularly cardiovascular diseases and osteoarthritis. In these cases, pharmacological treatment must be evaluated due to the risk of pharmacodynamic pDDIs (e.g., Ace inhibitors and NSAIDs) with a decrease in blood pressure control.

### 4.1. Proton Pump Inhibitors

The most common group of drugs involved in pDDIs were proton pump inhibitors, i.e., omeprazole and esomeprazole. The 2021 National Report on Medicines Use in Italy, prepared by AIFA’s Medicines Utilization Monitoring Centre (OsMed), documented that proton pump inhibitors represent a group of drugs commonly prescribed, and we reported that this prescription is common during the discharge period. Previously, we documented that omeprazole and esomeprazole are able to induce ADRs during hospitalization [4]. Now, we document that these drugs are involved in 198 pDDIs, even if we only recorded clinically relevant DDIs in 10 patients.

Interestingly, we documented that two patients with rheumatoid arthritis treated with methotrexate received a prescription of omeprazole. In these patients, an increase in methotrexate plasma levels was recorded due to the inhibition of the renal H+/K+ pump, leading to a decrease in methotrexate excretion. This interaction (D class, DIPS: 7) is common to all proton pump inhibitors (omeprazole, esomeprazole, lansoprazole, rabeprazole, and pantoprazole), so it is necessary to stop these drugs three days before methotrexate administration or change them to a histamine H2 receptor antagonist (e.g., famotidine, which does not affect drug metabolism and is free of antiandrogenic effects).

### 4.2. Statins

In this study, simvastatin and atorvastatin were involved in 159 pDDIs without clinical effects. However, in five patients discharged on atorvastatin, we documented the development of myalgia during coadministration with clarithromycin (C class, one patient) and sildenafil (D class, four patients).

These pDDIs are described in clinical settings [22,23,24,25] and are related to the inhibition of cytochrome P450 (CYP) 3A4. Therefore, in these patients, we suggest changing the type of statin (using statins not metabolized by CYP3A4, e.g., rosuvastatin, high potency, or pravastatin, low potency) or changing the type of antimicrobial drug (e.g., azithromycin) or phosphodiesterase-5 inhibitor (e.g., vardenafil). However, other compounds can modify the activity of CYP3A4, increasing (inhibitors) or reducing (inductors) the blood concentration of statins (atorvastatin, lovastatin, and simvastatin), and these must be considered during hospital discharge.

### 4.3. Clopidogrel

We documented the pDDIs between clopidogrel and omeprazole (*n* = 7) or esomeprazole (*n* = 1) in individuals who had been hospitalized probably related to the low effectiveness of clopidogrel. Clopidogrel is a prodrug that is bio-transformed in the liver via CYP2C19 into an active metabolite that binds irreversibly to the platelet ADP receptor P2Y12, preventing ADP-induced platelet aggregation. [26]. Omeprazole, lansoprazole, and esomeprazole inhibit CYP2C19, reducing the antiplatelet effect of clopidogrel [27].

Even if it could be a DDI, we are not able to demonstrate if this is a probable ADR because the clinical symptoms could be related to other gender-related factors, and no tests were performed to demonstrate a decrease in clopidogrel activity.

However, to reduce the risk of DDIs in older patients, we suggest using proton pump inhibitors without effects on CYP (e.g., pantoprazole). The same mechanism involves the DDIs of clopidogrel with fluoxetine (*n* = 5), and therefore, it would be prudent to use SSRIs that do not act on CYP3A4 (e.g., sertraline, citalopram, and escitalopram).

### 4.4. Antimicrobial Drugs

Usually, the risk of drug interactions during antimicrobial treatment is not well evaluated, due to the short duration of treatment (usually less than 7–10 days). Kusku et al. [28] documented, in a multicentric study, that quinolones, metronidazole, linezolid, and clarithromycin were responsible for 92% of the reported DDIs. Other authors documented that, in patients referred to intensive care units, the DDIs due to quinolones were associated with QT prolongation [29], suggesting particular attention during the prescription. Moreover, other authors reported that ciprofloxacin inhibits CYP1A2, with an increase in the blood concentration of substrate drugs (e.g., olanzapine and tizandine) and an increase in ADRs (e.g., QT prolongation and somnolence, respectively) [30,31].

In the present study, we documented 87 pDDIs in older patients with a prescription of ciprofloxacin or levofloxacin without clinical effects.

Only one patient treated with levofloxacin for acute exacerbations of chronic obstructive pulmonary disease was hospitalized for a QT prolongation due to a DDI with Lenvatinib (D class). Lenvatinib is an oral active tyrosine kinase inhibitor that represents a promising targeted therapeutic for several types of cancer [32]. Lenvatinib is metabolized by CYP1A1 and CYP3A4, so strong inducers or inhibitors of these CYPs could pose a risk of DDIs, potentially resulting in a reduction in anticancer efficacy or an increase in drug-related toxicity [33].

### 4.5. SSRI

A QT interval prolongation was reported in three patients hospitalized for a DDI between citalopram (*n*: 2) or escitalopram (*n*: 1) and Lenvatinib. The analysis of DDIs suggests that it is not related to a pharmacokinetic DDI but to a potentiation of their ADRs. In fact, Shah and Morganroth [34], reviewing literature data, documented that Lenvatinib can induce QT interval prolongation, left ventricular dysfunction, and hypertension. QT interval prolongation represents the risk of a pDDI that we recorded during the prescription of citalopram (and SSRIs) in patients treated with amitriptyline (C class). However, we also demonstrated a pDDI after the prescription of SSRIs in patients using NSAIDs, amitriptyline, triptans, or tramadol. SSRIs increase the levels of serotonin, and together with other drugs able to do so (tramadol and triptans), they can induce the development of serotonin toxicity (C and D classes, respectively). Nelson and Philbrick [35] documented the risk of an interaction between tramadol and SSRIs related to an increase in serotonin toxicity. Moreover, in this study, we documented an interaction (C class) between amitriptyline and tramadol. Even if this interaction could be related to a pharmacodynamic DDI, due to the increase in serotonin concentrations, it is also possible to have a pharmacokinetic DDI. In fact, the main metabolic pathway of tramadol is through the CYP 2D6 enzymes, which may be partially inhibited by amitriptyline, with an increase the in tramadol concentration and in serotonin effects. In this study, we documented the prescription of fluoxetine in patients treated with triptans for headache management. Although it is a D class DDI, we did not record any hospitalization in these patients, probably due to the low duration of treatment with triptans (e.g., sumatriptan, frovatriptan), though we are not able to demonstrate this.

Another pDDI recorded during the study was related to the prescription of paroxetine or fluoxetine (*n*: 6) in patients with an autoprescription of diclofenac for pain treatment. This is a C class DDI, and Anglin et al. [36], in a meta-analysis of 19 observational studies, reported an increase in gastrointestinal bleeding rates in patients using both SSRIs and NSAIDs compared with those in non-users (OR 4.25, 95% CI 2.82–6.42). More recently, Haghbin et al. [37], in a systematic review and network meta-analysis, documented an increased risk of gastrointestinal bleeding in patients treated with SSRI and NSAIDs, compared with that in patients treated with SSRI alone (36.9% vs. 22.8%, OR 2.14, 95% CI 1.52–3.02, *p* < 0.001) or with NSAIDs alone (40.9% vs. 34.2%, OR 1.49, 95% CI 1.20–1.84, *p* < 0.001), suggesting caution when administering NSAIDs and SSRIs concurrently.

### 4.6. NSAIDs and Pain Killers

Another cause of severe bleeding may be the association of NSAIDs with enoxaheparin due to pharmacodynamic mechanisms (C class). In this study, we documented this pDDI in eight patients, but we failed to document the development of ADRs. However, we suggest avoiding these drugs together. In the presence of coadministration, we suggest carefully monitoring the clinical conditions.

Finally, we need to pay attention during the pain treatment. In fact, we recorded two prescriptions of tramadol and three of tapentadol for pain management in patients using oxycodone (C class) that required careful observation. Moreover, we documented four prescriptions of tramadol and oxycodone in patients using at-home benzodiazepines (C class). In this study, these DDIs did not induce the development of ADRs. However, in these patients, respiratory function and comorbidities (e.g., chronic obstructive respiratory disease, overlap sleep syndrome) must be evaluated to reduce the risk of respiratory failure.

A statistical evaluation documented a significant correlation among pDDIs, age, number of drugs used, and sex (women), but we failed to document a correlation with gender because the study population was enrolled in the same country without differences with respect to country, race (all patients were white), economy (all patients were retirees and worked in public offices or in the countryside), or religion (all people were Catholics).

As previously reported, the number of drugs used increases the possibility of pharmacokinetic and pharmacodynamic drug interactions [6]. Regarding sex, Zucker and Prendergast [38] documented that women are exposed to higher blood drug concentrations and longer drug elimination times than men, and this could explain the difference in drug safety between the sexes. Even if we are not able to demonstrate the difference in drug concentrations between men and women (this is a retrospective study), our data suggest that women show a high risk, so careful monitoring is suggested for older women.

The present study has some limitations related to the design (data recorded on clinical records) and the use of software. In fact, since the use of computerized software is highly desirable, its value depends on both its sensitivity (in detecting DDIs) and accuracy (in assessing the type and severity of DDIs).

## 5. Conclusions

In conclusion, this study has documented that potential drug interactions represent a risk factor after hospital discharge. We think that an approach to the management of these pDDIs might be achieved through a clinical pharmacology consultation at the bedside before discharge that involves the caregiver and the general practitioner and using drug interaction software and therapeutic drug monitoring, leading to a reduction in the risk of new hospitalizations in this group of patients. However, it is important that physicians and general practitioners take their time in their discussions with caregivers and with patients, to carefully consider lowering doses and deprescribing where possible.

## Figures and Tables

**Table 1:** Morbidity in patients included in this analysis. Data are expressed as a percentage of included patients (*n* = 1772).

Disease	Percentage
Cardiovascular diseases (Blood hypertension, atrial fibrillation)	82.3
Osteoarthritis	79.5
Diabetes mellitus type 2	26.2
Depression	18.7
Impaired cognition, dementia	16.8
Gastroesophageal reflux disease	13.3
Urological diseases	12.8
COPD	8.4
Rheumatological diseases	6.9
Osteoporosis or osteopenia	4.8
Asthma	1.2
Hypothyroidism	1.1
Other	1.6

**Table 2:** The demographic characteristics of the included patients (*n* = 1772) with and without potentially inappropriate prescriptions (PIPs) were evaluated after hospital discharge. The data are expressed as percentages with respect to the included patients. * *p* < 0.01 women vs. men. § *p* < 0.01 women with PIPs vs. women without PIPs.

Characteristics	With PIP (*n*: 794)	Without PIP (*n*: 552)
Age (mean ± SD)	76.3 ± 7.3	76.8 ± 8.5
Men (%)	41.3	46.6
Women (%)	58.7 *§	53.4 *
Current smokers (%)	5.2	4.8
Overweight (%)	53.6	52.8
Chronic diseases (mean ± SD)	4 ± 2	5 ± 2

**Table 3:** Prescription of drugs inducing a potential drug interaction, during hospital discharge, in enrolled patients (*n* = 794). Data are expressed as a percentage respect to the enrolled patients and absolute number. SSRIs, serotonin selective reuptake inhibitors; SNRIs, serotonin noradrenalin reuptake inhibitors; NSAIDs, non-steroidal anti-inflammatory drugs.

Drugs	Percentage	Number
omeprazole and esomeprazole	25	199
atorvastatin and simvastatin	20	159
Clopidogrel	12.9	102
ciprofloxacin and levofloxacin	11	87
SSRIs	10.1	80
Opioids	9	71
SNRIs	6	48
Benzodiazepines	3	24
NSAIDs	3	24

**Table 4:** Potential drug interactions in enrolled patients (*n* = 794). The total number of patients using these drugs are in parentheses. SSRIs, serotonin selective reuptake inhibitors; SNRIs, serotonin noradrenalin reuptake inhibitors; NSAIDs, non-steroidal anti-inflammatory drugs.

Drugs Prescribed during Hospital Discharge	Drugs in Therapy
omeprazole and esomeprazole	citalopram (28), escitalopram (26), venlafaxine (25), tramadol (21), trazodone (12), aripiprazole (11), tizanidine (10), alfuzosin (9), domperidone (9), ciprofloxacin (9), amiodarone (9), vardenafil (8), levofloxacin (7), haloperidol (6), clopidogrel (5), moxifloxacin (4)
atorvastatin or simvastatin	esomeprazole (59), amlodipine (16), Sacubitril Valsartan (14), sitagliptin (12), ranolazine (9), amiodarone (9), tadalafil (8), dronedarone (6), warfarin (6), sildenafil (6), clopidogrel (3), diltiazem (3), ticagrelor (3), carbamazepine (2), everolimus (2), domperidone (1)
Clopidogrel	omeprazole (22), esomeprazole (18), lansoprazole (16), rosuvastatin (12), repaglinide (9), fluoxetine (6), paroxetine (5), tramadol (5), tapentadol (4), venlafaxine (3), amiodarone (2)
ciprofloxacin and levofloxacin	escitalopram (14), duloxetine (9), venlafaxine (6), tramadol (4),
	omeprazole (10), metformin (9), simvastatin (7), betamethasone (3), paroxetine (3), fluoxetine (3), warfarin (2), furosemide (2), propafenone (1), ranolazine (1), zolpidem (1)
	diclofenac (6), ibuprofen (4), dutasteride (2)
fluoxetine and paroxetine	rivaroxaban (6), apixaban (4), dabigatran (4), diclofenac (6), clopidogrel (6), tizanidine (4), tramadol (4), triazolam (3), frovatriptan (3), furosemide (2), trazodone (2), oxycodone (2), triazolam (2), amiodarone (2), almotriptan (1), eletriptan (1)
citalopram and escitalopram	omeprazole (7), tramadol (3), tizanidine (3), apixaban (2), trazodone (2), diclofenac (2), ibuprofen (2), ketoprofen (2), amitriptyline (2), buprenorphine (2), almotriptan (1)
Tramadol	omeprazole (7), gabapentin (7), paroxetine (6), amitriptyline (6), duloxetine (4), fluoxetine (2), oxycodone (2), alprazolam (1), diazepam (1)
Tapentadol	citalopram (6), escitalopram (3), paroxetine (3), oxycodone (3), almotriptan (2), trazodone (2), sertraline (2), risperidone (2)
Oxycodone	escitalopram (6), paroxetine (3), Triazolam (2), gabapentin (1)
Venlafaxine	omeprazole (9), esomeprazole (9) flecainide (6), ciprofloxacin (5), azithromycin (4), zolmitriptan (2) buprenorphine (1)
Duloxetine	trazodone (3), clobazam (2), rizatriptan (2), naproxen (2), etoricoxib (1)
Diazepam	omeprazole (16), tramadol (4), tapentadol (2), olanzapine (2)
alprazolam and triazolam	tramadol (2)
diclofenac and ketorolac	enoxaparin (8), venlafaxine (5), valsartan (5), furosemide (4), levofloxacin (2)

**Table 5:** Twenty-four drug interactions recorded in the study requiring hospitalization using the Drug Interaction Probability Scale (DIPS). Drug 1 is the drug prescribed during the discharge; Drug 2 is the drug used at home.

Drug 1	Drug 2	ADRs	Mechanism	Action	N Patients	DIPS Score
citalopram 20 mg once per day	lenvatinib 10 mg once per day	Increase in heart rate	QT interval prolongation	Change citalopram to duloxetine	2	6
escitalopram 20 mg once per day	lenvatinib 10 mg once per day	Increase in heart rate	QT interval prolongation	Change escitalopram to duloxetine	1	6
levofloxacin 500 mg once per day	lenvatinib 10 mg once day	Increase in heart rate	QT interval prolongation	Change levofloxacin to piperacillin/tazobactam	1	6
clopidogrel 75 mg once per day	omeprazole 40 mg once per day	Change in clopidogrel activity	Reduced liver activation of clopidogrel	Change omeprazole to pantoprazole	7	6
clopidogrel 75 mg once per day	esomeprazole 40 mg once per day	Change in clopidogrel activity	Reduced liver activation of clopidogrel	Reduce the dosage of esomeprazole to 10 mg	1	6
clopidogrel 75 mg once per day	fluoxetine 20 mg daily	Change in clopidogrel activity	Reduced liver activation of clopidogrel	Change fluoxetine to sertraline	5	6
omeprazole 20 mg daily	Methotrexate	Increased methotrexate toxicity	Reduced renal secretion of methotrexate	Stop omeprazole three days before the administration of methotrexate	2	7
atorvastatin 20 mg daily	sildenafil 20 mg	Muscular pain	CYP3A4 inhibition	Stop sildenafil change to vardenafil	4	6
atorvastatin 20 mg daily	clarithromycin 500 mg every 12 h	Muscular pain	CYP3A4 inhibition	Change clarithromycin to azithromycin	1	6

## Data Availability

The data presented in this study are available on request from the corresponding author. The data are not publicly available due to privacy.
